# Pennsylvania State Veterinary Medical Association

**Published:** 1901-01

**Authors:** C. J. Marshall

**Affiliations:** Recording Secretary


### PENNSYLVANIA STATE VETERINARY MEDICAL ASSOCIATION.

(Concluded from page 784, December number.)

*Reports of County Secretaries.*

Reports were made covering the work of counties and the well-being of the profession: Beaver County, Dr. Buckham; Crawford County, Dr. C. C. McLean; Erie County, J. B. Jones; Jefferson County, F. F. Hoffmann; Lawrence County, E. C. Porter; Mercer County, A. W. Weir; Warren County, M. J. Chrisman; Venango County, G. B. Jobson.

For Berks County, a written report was read from Dr. W. S. Phillips, of Reading: The livestock in general have been doing well through the exceptionally hot weather. There was a slight epidemic of pink-eye for some months, which has passed away. Of late there has been more attention paid to the raising of colts than for some years previously, quite a number of well-bred ones being foaled in our vicinity. Horses bring higher prices, and I do not think there will be any decline in the prices. During the last six weeks there have been one hundred and ten ranch horses brought here, which have been disposed of at fair prices, some well-bred ones being among them.

For Montgomery County, a report was read from Dr. S. D. Larzelere: As far as can be learned, the past summer has been both busy and satisfactory for the veterinary practitioner in this county; the work has been of a general character, not especially marked by any particular outbreak of disease. Among the diseases most prevalent I may mention influenza, which prevailed to a greater extent than for several years; the cases of this disease were of the average severity, with occasional fatal complications. Distemper certainly did not appear in above the average number of cases. Cerebro-spinal meningitis continues to occur in various localities, with the usual number of fatalities. Tuberculosis in cattle surely appears to be diminishing in amount; the operation of the State Livestock Sanitary Board has certainly done much to rid the county of the worst centres of infection, so that while occasional cases are met, yet the discovery of large herds badly and extensively diseased has become rare as compared with two years ago. The general public is becoming still better informed concerning the disease, and consequently approves the measures taken to control it. The growth of knowledge in this direction is most gratifying and insures for the future general coöperation in the fight against this common scourge. The dairymen of this section have received rather better remuneration for their labors than during the summer of 1899. The improvement in dairy methods has been noticeable; the use of the silo is increasing, and improved machinery and dairy appliances have had greater sale than ever before in this locality. Organization among farmers and dairymen has progressed considerably. The market price of horses and cattle continues high, yet the sales do not appear to have been below the average. The use of the bicycle appears to have somewhat diminished; automobile carriages are used to a very slight extent.

Adjournment for lunch, furnished by the local Committee of Arrangements, Dr. J. B. Irons, Chairman. The lunch proved very enjoyable, and added much to the pleasure of the visitors.

AFTERNOON SESSION.

The afternoon session was convened at 2.30. Dr. W. S. Phillips, of Reading, Pa., forwarded a paper on "Tumor on Shoulder," which was read by the Secretary and fully discussed by Drs. Emery, Bryce, Phillips, and Hoskins.

Dr. Hoskins read a paper on "Some Aspects of Future Legislation."

Dr. Jobson spoke of the importance of pushing the Army Legislation bill and urged the necessity of seeing our Congressmen and Senators; also the fact of Mr. Sibley pledging his support.

Dr. Pearson spoke of an attempt to pass two laws through the Legislature—one in regard to disposal of animals dead of infectious disease, the other in regard to the destruction of rabid dogs without the consent of the owners. He says these two laws are important. Under the present law a man cannot be compelled to destroy the carcass of an animal dead of an infectious disease; it has to be done by and through the State authorities. And as to the law in regard to rabies, if the owner cannot be found the animal cannot be destroyed, but simply quarantined. He also spoke about making too many la ws which were not enforced.

Dr. Hoskins was asked what he meant by a re-registration. When the law first went into effect fees were required; everybody registered, enriching the prothonotary. A re-registration would eliminate this class. Money should be paid into the board.

Dr. Hoskins emphasized what Dr. Jobson said in reference to army legislation and the importance of getting every Congressman promise to support the bill before the next session.

Dr. Pearson offered a resolution in reference to Drs. Klein and McNeall being appointed to instructorships in Ames (Iowa) Agricultural College. Resolution was unanimously adopted.

Dr. Jobson was called on the subject of "Cattle Inspection and the Tuberculin Test." After a few preliminary remarks he read his paper, which was very interesting and instructive.

Dr. Ranck agreed with Dr. Jobson about testing the cases there was some reason for not testing at the same time with others of the herd.

Dr. Pearson spoke of the change that the State Livestock Sanitary Board had made in the last two years in reference to testing cows about to calve; also spoke of the difficulty in appraising diseased cows. They should be appraised as they appear without the tuberculin test. He spoke of the mistaken opinion of some that the tuberculosis question is dying out, and the rejoicing on this account; but more work is now being done than ever. Simply the fighting of it is dying out. Many questions were asked on this subject and cases cited.

Address given by Dr. Pearson on "Abortion." Contagious abortion only; not traumatic. Enzoötic in many places, and can find it almost constantly in stock-raising districts. Bang discovered the germ; small, but definite amount of oxygen needed, and grows only at a certain depth in gelatin; it grows equally well in uterus. Propagated from cow to cow by the bull. Long period of incubation—*i. e.*, a few weeks or months. Recent work by Prof. Ostertag (German), eight cows were served by a bull, two cows aborted. Had bull serve these cows, and only those served by this bull aborted. Cows pregnant placed between infected cows were not infected, which shows the bull carries contagion. Period of incubation may be six to seven months. Treatment: Sheath of bull irrigated three times a day for four days (0.5 per cent. solution of lysol), then bull served healthy cows; result, no abortion. Lysol solution injected in the uterus; results good. Carbolic acid treatment also followed.

Susquehanna County, by Dr. Tower, reported similar treatment; results good.

Dr. Porter gave his report of the accomplishments of the meeting at Detroit. Clinics were held each morning at the hospital of Dr. S. Brenton. Citing of filling cavity, after removing tooth, with gutta-percha; oöphorectomy on a mare; ovariotomy on the bitch was performed (flank operation) by Dr. W. L. Williams, of Ithaca, N. Y.; Dr. Baker, of Chicago, Ill., operated on a bursitis as a result of speedy cut.

*Resolutions.*

The following resolutions were adopted:

We, the undersigned members of the Pennsylvania State Veterinary Medical Association, in convention assembled at Erie, Pa., September 18, 1900, do most cordially second the efforts of our colleagues in the State of New Jersey in soliciting the place of meeting of the American Veterinary Medical Association for Atlantic City in 1901;

Believing that the time is propitious for a successful meeting in that State, which for many years held a strong place in numbers and influence in the National Association, and that its large coterie of veterinarians today, their splendid work in forming one large, harmonious State organization, and the attraction of their wonderful seaside resort, commend most favorably this city for 1901. This resolution and petition was signed by S. J. J. Harger, Philadelphia, Pa.; W. Horace Hoskins, Philadelphia Pa.; George B. Jobson, Franklin, Pa.; C. Courtney McLean, Meadville, Pa.; E. C. Porter, New Castle, Pa.; Leonard Pearson, Philadelphia, Pa.; F. F. Hoffman, Brookville, Pa.; C. J. Marshall, Philadelphia, Pa.; C. Z. Laberge, Beaver Falls, Pa.; A. W. Weir, Greenville, Pa.; H. Emery, Pittsburg, Pa.; J. B. Irons, Erie, Pa.; W. H. Fry, Pine Grove Mills, Pa.; M. J. Chrisman, Sugar Grove, Pa.; A. W. Bradley, Bethlehem, Pa.; John Gobin, North Collins, N. Y.; E. G. Britton, Franklin, Pa.; James Buckham, Beaver Falls, Pa.; R. M. Phelan, Sharon, Pa.; E. E. Bittles, New Castle, Pa.; W. P. Bagnall, Warren, Pa.; E. M. Ranck, Philadelphia, Pa.; J. R. Phillips, Northeast, Pa.; W. A. Meredith, Corry, Pa.; A. E. Cunningham, Cleveland, Ohio; J. Bryce, Erie, Pa.

WHEREAS, Our meeting in Erie has been a peculiarly successful one, of much interest and importance to the welfare of the profession; and

WHEREAS, This being largely due to the efforts of the local committee and the very active work of Chairman J. B. Irons; therefore, be it

*Resolved*, That we extend our sincere thanks to those who have contributed so much to the pleasure and profit of this semi-annual gathering.

WHEREAS, Two of our colleagues, Dr. L. A. Klein and Dr. J. H. McNeall, have been elected to professorships in the Veterinary Department of the Iowa State College; and

WHEREAS, Drs. Klein and McNeall were among the most active and progressive veterinarians in Pennsylvania during their temporary residence in this State, and were always interested in and ready to work for the advancement of veterinary science and professional progress; be it

*Resolved*, That we regret that Drs. Klein and McNeall have decided not to remain permanently in this State; that we congratulate them on this well-deserved recognition of their merit, and that our best wishes are extended to them in their new field of labor; be it further

*Resolved*, That we commend our former associates to the veterinarians of the State of their adoption, with the assurance that they have gained two colleagues who will honor their profession and aid all worthy causes in which it is interested; be it further

*Resolved*, That a copy of these resolutions shall be sent officially from this Association to the State Veterinary Society of Iowa.

Committee on Resolutions: Leonard Pearson, C. J. Marshall, and W. Horace Hoskins.

C. J. MARSHALL,
Recording Secretary.

